# Effect of irradiation with intravascular laser on the hemodynamic variables of hypertensive patients: Study protocol for prospective blinded randomized clinical trial

**DOI:** 10.1097/MD.0000000000015111

**Published:** 2019-04-05

**Authors:** Adriana Paula Jordão Isabella, Jéssica Tayane Costa Silva, Tamiris da Silva, Maria Fernanda Setúbal Destro Rodrigues, Anna Carolina Ratto Tempestini Horliana, Lara Jansiski Motta, Sandra Kalil Bussadori, Christiane Pavani, Daniela de Fátima Teixeira da Silva

**Affiliations:** a PhD student of Post Graduate Program in Biophotonics; b Department of Health, Bachelor of Nursing, Nove de Julho University (UNINOVE); c Post Graduate Program in Rehabilitation Sciences; d Post Graduate Program in Biophotonics, Nove de Julho University (UNINOVE), São Paulo, Brazil.

**Keywords:** arterial hypertension, cardiovascular system, low intensity laser therapy, photobiomodulation, systemic effect

## Abstract

**Background:**

High blood pressure (HBP) is a multifactorial clinical condition, with a high morbidity and mortality rate and low rates of control. Due to its high prevalence, it is necessary to search for methods which aim to improve the quality of life of hypertensive patients. Studies have shown that low level laser therapy (LLLT) is capable of inducing a photobiological response within the cells which modifies the micro and macrovascular response; this accompanies evidence showing the systemic effects of intravascular laser irradiation of blood (ILIB). In the hypothesis that the use of LLLT can influence blood pressure levels, and perhaps facilitate adherence to treatment, this study aims to present a clinical research protocol with the goal of determining the effect of photobiomodulation in relation to changes in the hemodynamic parameters of hypertensive and normotensive patients.

**Method:**

Forty-four participants, frequent attendees of an ambulatory university clinic, will be subdivided into 4 groups, and then submitted to protocol sessions of ILIB. The technique is noninvasive and consists of a laser which is attached to a bracelet which has been specifically developed for the light beam to be transcutaneously carried over the radial artery. Before the procedure, at the end of the photobiomodulation cycles, and 1 month after the end of therapy, blood samples will be collected for the evaluation of C-reactive protein, interleukin 6, and nitric oxide, to be analyzed by immunoturbidimetric, ELISA, and Griess reactions, respectively.

**Analysis of results:**

Results will be analyzed using descriptive and inferential statistics and will be compiled into tables and/or graphs, with the help of SPSS version 24.0 with the adopted significance level for all tests being α = 0.05.

**Discussion:**

The treatment of HBP involves both pharmacological and nonpharmacological therapy. Animal studies with photobiomodulation have previously shown hypotensive effects. Gaps in the literature exist regarding the applicability of this nonpharmacological therapy in humans. This study aims to consider the possibility of offering nonpharmacological therapy to hypertensive patients with the goal of increasing adherence to the treatment as well as minimizing morbidity and mortality caused by hypertension.

## 1 Introduction

High blood pressure (HBP) is a clinical condition of multifactorial origin and metabolic alterations. Considered a global public health problem unsatisfactorily controlled, it represents the most prevalent vascular disease in the world. Characterizing one of the principle modifiable risk factors and main underlying cause of death due to cardiocerebrovascular disease, its prevalence is also reflected in high hospital admission rates and its high costs to public coffers.^[1–6]^

Blood pressure (BP) control is one of the most complex physiological functions of the organism. It depends on the integration of the cardiovascular, renal, neural, and endocrine systems, ^[7,8]^ acting together to maintain control of cardiac output and peripheral vascular resistance. The manifestation of hypertension is therefore necessarily related to changes in these parameters. ^[9,10]^

Systemic vascular resistance is mainly regulated in the regions of the arterioles and is influenced by neural and hormonal factors. The normality of vascular tone comes from the balance between circulating factors that induce vasoconstriction (such as angiotensin II and catecholamines) and vasodilation (such as kinins, prostaglandins, and nitric oxide [NO]). Other factors are also involved in this process, such as pH, hypoxia, and neural interactions (α and β adrenergic systems).^[11]^

Endothelial lesions result in an inflammatory response, with the performance of various cell types (lymphocytes, monocytes, platelets, and smooth muscle cells), ^[12]^ causing endothelial cell dysfunction, vascular wall stiffening, and atherosclerosis plaque formation.^[13]^ These endothelial lesions and complications are confirmed by inflammatory markers that result in endothelial activation, such as C-reactive protein (CRP) and interleukin 6 (IL-6), ^[14,15]^ this last 1 correlating positively with systolic and diastolic pressures. Acting in conjunction with these for BP control, the kidney also produces several vasorelaxative and antihypertensive substances (such as prostaglandins and NO), which balance the vasoconstricting action of angiotensin II.^[8,9,10]^

Currently, the treatment of hypertension is based on a nonpharmacological and pharmacological combination, aimed at implanting strategies that may reverse or prevent the progression of these factors, as well as the prescription of drugs with vasodilatory actions, beta blockers, and diuretics, leading to a decrease in BP.^[11,16]^ It should be emphasized that the use of drugs can cause the manifestation of signs and symptoms which are the result of adverse or collateral effects. This in turn may influence the patient's adherence to treatment, which consists in the acceptance of and obedience to what was prescribed and directed by the attending healthcare professional.^[17]^ Non-adherence is identified as the main cause of uncontrolled arterial hypertension ^[18]^ therefore it becomes imperative to find methods which facilitate this process with the purpose of promoting improvement in the quality of life of these patients.

Low intensity laser therapy, or photobiomodulation, is capable of inducing a photobiological response inside the cells; activating the production of adenosine triphosphate (ATP), NO, and reactive oxygen species; and altering sodium–potassium pumps and calcium channels in cell membranes, ^[19]^ as well as proving to be an efficient, noninvasive, low cost, safe tool.

Among the different methods of photobiomodulation, intravascular laser irradiation of blood (ILIB) has been shown to prompt systemic effects. ILIB has been studied since 1981 by Soviet scientists; it was developed for the treatment of cardiovascular diseases with evidence of improved blood rheological properties and microcirculation as well as reduction in the infarct area, cardiac arrhythmias, and sudden death. ^[20]^

In view of the above, the verification of the effects of systemic photobiomodulation on the control of BP in humans shows itself to be a current, relevant, promising area of study, principally in order to prevent patients’ health problems and the consequent high cost to public health, along with minimizing the gaps in the literature in this area.

Will be a study single-center, controlled, randomized, blinded clinical trial whose general objective will be to evaluate the effects of systemic photobiomodulation on the hemodynamic variables of hypertensive patients (the primary outcome variable will be BP values). Besides, will be analyzed the dosages of IL-6, CRP, and NO.

## 2 Methodology/design

### 2.1 Description of the proposed study

It is a single-center, controlled, randomized, blinded clinical trial outlined in accordance with the criteria set out by the SPIRIT statement. The sample will consist of 44 participants in medical follow-up at the Nove de Julho University Integrated Outpatient Healthcare Clinic located in the city of São Paulo, Brazil, from March 2019 to August 2019.

After verbal and written explanation, individuals who agreed to participate in the study will sign the Free and Informed Term of Consent already approved by the Nove de Julho University's Research Ethics Committee registered under CAAE 85714318.3.0000.5511.

### 2.2 Clinical trial registry

The protocol for this study was registered in the Brazilian Registry of Clinical Trials—ReBEC (RBR-7n55nz), first received in February 2019, http://www.ensaiosclinicos.gov.br/rg/?q=RBR-7n55nz.

### 2.3 Sample size

The determination of the number of patients necessary was calculated using G ∗ Power software (version 3.1.9.2, Franz Faul, Universität Kiel, Germany), in which the values for the mean and standard deviation of the primary outcome variable (systolic or diastolic BP) came from the article by Pereira et al.^[21]^ The calculation was performed using the ANOVA repeated measures, with a significance of 5% and power of test at 95%. According to sample calculation, 44 hypertensive patients will be divided into 4 groups with 11 individuals each.

### 2.4 Inclusion criteria

Hypertensive people between 30 and 80 years old; officially diagnosed with HBP by a medical professional; in stages I and II; and evidenced during ambulatory BP and hypertension monitoring with at least 4 weeks of drug therapy.

### 2.5 Exclusion criteria

Patients who present hypotension before photobiomodulation; pregnant women; glaucoma patients; carriers of electronic implants, such as cardiac pacemakers; epilepsy; seizures; history of neoplasias; and patients with photosensitivity.

### 2.6 Group composition and randomization: (recruitment and randomization)

Recruitment will be carried out respecting Resolution 466/2012, which regulates human research in Brazil. The patients recruited will have their multiprofessional charts analyzed for demographic and descriptive characterization of the sample.

Nursing consultations in the form of a home visit, or not—at the discretion of the participant, will be scheduled to give healthcare guidance, for an initial interview, completion of the clinical file, and realization of hemodynamic and anthropometric measurements, as well as blood collection and photobiomodulation interventions.

For the application of the photobiomodulation, the participant will place a bracelet where the tip of the coupled laser equipment will be positioned on the radial artery.

We will use the randomization mechanism provided by Sealed Envelope Ltd. (London, UK)^[22]^ Participants will be randomized into blocks of 8 individuals, comprising 4 study groups. They will be identified by sequential numbers, according to the order of recruitment. Immediately before starting treatment, the researcher will look at the randomized list and, using their identification number, verify which group the participant belongs to. The patient will not know to which group he or she has been allocated; only the researcher who will do the irradiations will know. The analysis of the data will be performed by a third person who will also not know group allocations.

**Figure 1:**
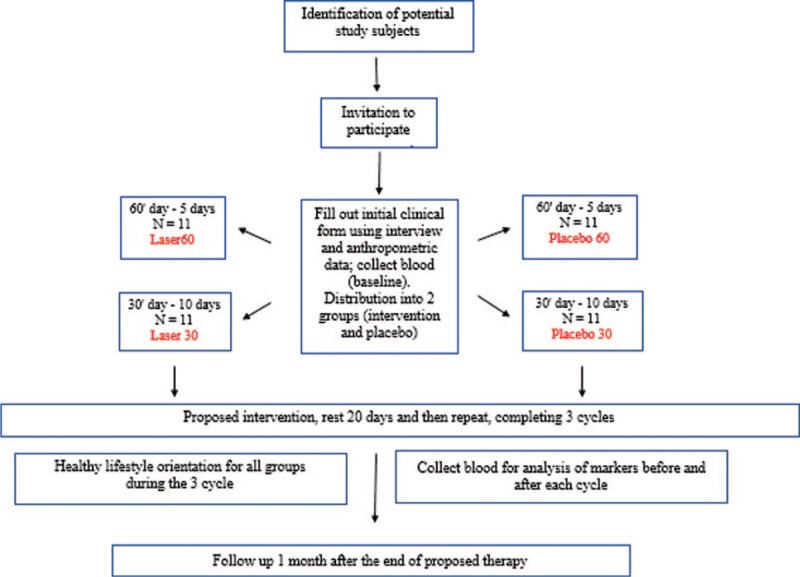
Flowchart describing study design, sample composition, and experimental protocol.

A schematic diagram of time schedule of enrolment and interventions of the research groups is summarized in Figure 1.

Research will be carried out to evaluate the effects of photobiomodulation using 2 ILIB protocols established by the manufacturer of the laser equipment: 60 minutes daily exposure time (Laser 60) and 30 minutes daily exposure time (Laser 30), with respective placebo groups (Placebo 60 and Placebo 30):

- Laser 60 (N = 11): will be submitted to the conventional drug treatment previously instituted and ILIB sessions for 60 minutes daily for 5 days, to be repeated after 20 days and, after completing 3 cycles, the patients will be reevaluated 1 month after the end of the proposed therapy.
- Placebo 60 (N = 11): will be submitted to the conventional drug treatment previously instituted and placebo sessions of ILIB for 60 minutes daily for 5 days, to be repeated after 20 days and, after completing 3 cycles, the patients will be reevaluated 1 month after the end of the proposed therapy. The placebo will be performed by placing a beam obturator at the laser output, so that all noise from the equipment will remain the same as that of the Laser 60 group, but no radiation will be delivered to the target.
- Laser 30 (N = 11): will be submitted to the conventional drug treatment previously instituted and ILIB sessions for 30 minutes daily, for 10 days, being repeated after 20 days and, after completing 3 cycles the patients will be reevaluated 1 month after the end of the proposed therapy.
- Placebo 30 (N = 11): will be submitted to the conventional drug treatment previously instituted and ILIB sessions for 30 minutes daily, for 10 days, being repeated after 20 days and, after completing 3 cycles, the patients will be reevaluated 1 month after the end of the proposed therapy. The placebo will be performed with the same obturator method mentioned previously.

During the study, strategies will be follow to improve adherence to intervention protocols, as nursing consultations in the form of a home visit, or not—at the discretion of the participant. In addition, patients who have a prescription for drug therapy should not suspend it. All patients, regardless of pharmacological or nonpharmacological use, will receive guidelines for the nonpharmacological treatment of hypertension, focusing on the need to maintain a healthy lifestyle, minimizing risk factors for hypertensive disease. These orientations include directions for healthy eating with decreased levels of sodium, fat, and sugar; regular physical exercise with the supervision of a professional; the need for quality sleep of at least 6 to 8 hours; abandonment or diminution of smoking; and the need for stress-minimizing leisure. Patients taking medication will have guidelines regarding the adequate use of medications, respecting the dose and prescribed schedule, reinforced.

Patients have autonomy for discontinue interventions and in this case, they will be replaced, but the same allocation will be remaining. Any outcome data collected from patients will be disconsidered and the participants will remain in ambulatory care.

### 2.7 Outcomes

Measurements of BP (primary outcome) and heart rate will be performed before, during (every 5 minutes), and at the end of each session, being measured again in the follow-up.

Blood will be collected from all participants to measure the dosage of IL-6, CRP, and NO. Collection will be performed at the beginning of the study and before each cycle (BASELINE—B1, B2, B3), at the end of each cycle of photobiomodulation (END—E1, E2, E3) and 1 month after the end of the proposed therapy (FL-UP). In total, there will be 7 samples from each patient.

Serum samples will be collected in two 10 mL dry tubes and will be processed in a centrifuge at 1000 rpm for 5 minutes and the plasmas at the top of the tubes will be withdrawn and centrifuged again to obtain clear plasmas. The samples will be stored in a freezer at −80° C until the analyses are performed.

The CRP analysis will be performed by means of the immunoturbidimetric test, which allows the quantification of the CRP concentration present in the sample when following the protocol established by the kit manufacturer. IL-6 analysis will be performed via the ELISA method with the use of a specific kit (Human IL-6 ELISA MAX Deluxe, BioLegend, San Diego). The concentration of NO will be determined using a specific kit (Total NO and Nitrite/Nitrite Parameter Assay Kit), where nitrite/nitrate concentration will be determined via the Griess reaction after enzymatic reduction of nitrate in nitrite with the nitrate reductase enzyme in a reduction solution. This procedure will be in accordance with those recommended by the manufacturers.

### 2.8 Procedure for data collection, blinding, and photobiomodulation

A researcher will generate the allocation sequence using the Sealed Envelope Ltd. An examiner will enroll and assign participants to interventions, performing anthropometric assessments, measurement of hemodynamic variables, and blood collection. Other one will be trained to perform the photobiomodulation and will process the blood samples. Other researcher will perform the statistical analyzes of the results without having participated in the collection of data or the application of the procedures. In this way, the last researcher and the patients will be blind. Only after the end of the trial, the participants will know the allocation and results.

A data collection instrument was developed to contain the information needed to achieve the proposed objectives. The variables will be addressed in categories: characterization of the sample, evaluation of risk factors for cardiovascular disease, anthropometric data, data referring to BP measurements, heart rate, biochemical markers before and after photobiomodulation, as well as approach to the use of medicines, diet, and complementary information.

The photobiomodulation will be applied using the Therapy XT laser equipment, brand DMC, Brazil, following the parameters described in Table 1.

**Table 1:**
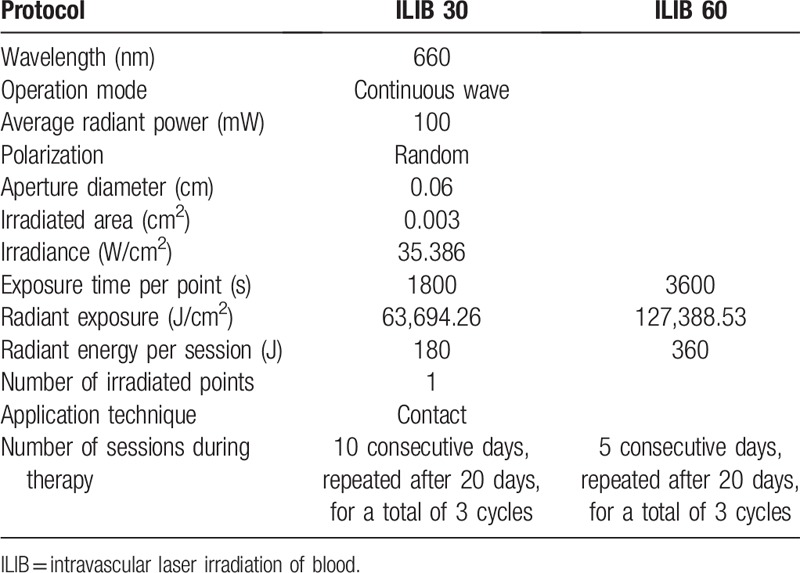
Dosimetric parameters proposed for the study.

It is considered that this study has minimal risks since, considering the hypothesis of BP reduction due to vasodilatation caused by photobiomodulation, subjects may present BP decrease with possible presence of clinical signs such as lipothymia, vertigo, complaint of malaise by the patient. If this happens, therapy will be stopped immediately and Trendelenburg maneuvers will be performed to increase venous return and BP.

### 2.9 Analysis and publication of data

After data collection, the data will be grouped into categories using Microsoft Office Excel 2007 (Redmond). The data will be analyzed using descriptive and inferential statistics and compiled into tables and/or graphs with the help of SPSS version 24.0 (IBM, New York). If the data are normal, parametric tests will be used to test inter and intragroup differences.

If they are not normal, nonparametric tests will be used. The level of significance adopted for all tests will be α = 0.05. The data will be protected in a spreadsheet and in a cloud under the care of the researchers. After the study, the same will be presented for publication in the form of a scientific article, where readers will have access to the results obtained in this research.

## 3 Discussion

Studies have demonstrated, both in vitro and in vivo, the control of inflammatory process and oxidative stress, as well as the production of NO with vasodilator and angiogenic action. Therefore, LILT can biomodulate factors that are closely related to endothelial dysfunction. ^[23–26]^

In a study carried out with sublingual laser application in hypertensive pregnant women, Madi^[27]^ observed that systemic vascular resistance and, consequently, systolic, diastolic, and mean arterial pressure, suffered a statistically significant decrease following photobiomodulation.

Weber et al^[28]^ described the different effects and mechanism of action of ILIB, including: anti-inflammatory effects that improve the immunological activity of blood; positive influence on rheological properties of the blood including vasodilatation, decreasing aggregation of thrombocytes, and a better deformability of the erythrocytes, thus resulting in a better supply of oxygen and with that a decrease in the partial pressure of carbon dioxide, such findings being relevant for the healing of wounds; improvement of hypoxia and tissue normalization with an increase in the synthesis of ATP occurring with the normalization of the cellular membrane potential; as well as effects of increased NO release from monocytes.

In Russian university clinics the use of intravascular irradiation is used to avoid thromboembolic complications and to improve the healing of postoperative wounds, bringing together the analgesic, spasmolytic, and sedative effects specific to the laser. There are additional reports of the benefits of such therapy in cases of chronic glomerulonephritis, for the improvement of inflammatory parameters in acute pyelonephritis and necrotizing pancreatitis, as well as the use of ILIB in obstetrics and gynecology to stimulate uteroplacental blood exchange and as a prophylaxis and therapy for inflammation of internal genitalia. ^[28]^

Despite promising results, clinical studies with an adequate level of scientific evidence are needed to fill the gap found in regards to the effects of systemic irradiation. Thus, it is expected that the present work will contribute to the scientific community, providing the first step to understanding the systemic effects caused by irradiation of the radial artery during the treatment of hypertension.

## Acknowledgments

The authors would like to thank the University Nove de Julho (UNINOVE) for the availability of laboratories and volunteers.

The authors would like to thank the Brazilian funding agency: scholarship of Jéssica Tayane Costa Silva #2018/19615-9, São Paulo Research Foundation (FAPESP).

## Author contributions

Conceive and design the study: A.P.J.I., J.T.C.S., D.F.T.S., A.C.R.T.H., L.J.M. Perform the experiment: A.P.J.I., J.T.C.S., T.S., M.F.S.D.R. Analyze the data: A.P.J.I., J.T.C.S., T.S., S.K.B. Perform the statistical analysis: D.F.T.S. and C.P. Write the paper: A.P.J.I., J.T.C.S., D.F.T.S.

**Conceptualization:** Adriana Paula Jordão Isabella, Daniela Fátima Teixeira Silva.

**Data curation:** Adriana Paula Jordão Isabella, Christiane Pavani, Daniela Fátima Teixeira Silva.

**Formal analysis:** Adriana Paula Jordão Isabella, Maria Fernanda Setúbal Destro Rodrigues, Daniela Fátima Teixeira Silva.

**Funding acquisition:** Daniela Fátima Teixeira Silva.

**Investigation:** Adriana Paula Jordão Isabella, Jéssica Tayane Costa Silva, Anna Carolina Ratto Tempestini Horliana, Lara Jansiski Motta, Daniela Fátima Teixeira Silva.

**Methodology:** Adriana Paula Jordão Isabella, Jéssica Tayane Costa Silva, Tamiris da Silva, Maria Fernanda Setúbal Destro Rodrigues, Anna Carolina Ratto Tempestini Horliana, Lara Jansiski Motta.

**Project administration:** Adriana Paula Jordão Isabella, Daniela Fátima Teixeira Silva.

**Resources:** Adriana Paula Jordão Isabella, Sandra Kalil Bussadori.

**Software:** Christiane Pavani, Daniela Fátima Teixeira Silva.

**Supervision:** Adriana Paula Jordão Isabella, Daniela Fátima Teixeira Silva.

**Validation:** Adriana Paula Jordão Isabella, Maria Fernanda Setúbal Destro Rodrigues, Daniela Fátima Teixeira Silva.

**Visualization:** Adriana Paula Jordão Isabella, Sandra Kalil Bussadori, Daniela Fátima Teixeira Silva.

**Writing – Original Draft:** Adriana Paula Jordão Isabella, Daniela Fátima Teixeira Silva.

**Writing – Review & Editing:** Adriana Paula Jordão Isabella, Daniela Fátima Teixeira Silva.

Daniela Fátima Teixeira Silva orcid: 0000-0002-7228-6146.
